# Non-random pairing of CD46 isoforms with skewing towards BC2 and C2 in activated and memory/effector T cells

**DOI:** 10.1038/srep35406

**Published:** 2016-10-14

**Authors:** Aida S. Hansen, Bettina B. Bundgaard, Bjarne K. Møller, Per Höllsberg

**Affiliations:** 1 Department of Biomedicine, Aarhus University, DK-8000 Aarhus C, Denmark; 2 Department of Clinical Immunology, Aarhus University Hospital, DK-8200 Aarhus N, Denmark

## Abstract

CD46 is a glycoprotein with important functions in innate and adaptive immune responses. Functionally different isoforms are generated by alternative splicing at exons 7–9 (BC and C isoforms) and exon 13 (CYT-1 and CYT-2 isoforms) giving rise to BC1, BC2, C1 and C2. We developed a novel real-time PCR assay that allows quantitative comparisons between these isoforms. Their relative frequency in CD4^+^ T cells from 100 donors revealed a distribution with high interpersonally variability. Importantly, the distribution between the isoforms was not random and although splicing favoured inclusion of exon 8 (BC isoforms), exclusion of exon 8 (C isoforms) was significantly linked to exclusion of exon 13 (CYT-2 isoforms). Despite inter-individual differences, CD4^+^ and CD8^+^ T cells, B cells, NK cells and monocytes expressed similar isoform profiles intra-individually. However, memory/effector CD4^+^ T cells had a significantly higher frequency of CYT-2 when compared with naïve CD4^+^ T cells. Likewise, *in vitro* activation of naïve and total CD4^+^ T cells increased the expression of CYT-2. This indicates that although splicing factors determine a certain expression profile in an individual, the profile can be modulated by external stimuli. This suggests a mechanism by which alterations in CD46 isoforms may temporarily regulate the immune response.

CD46 is a cell surface glycoprotein with regulatory functions in both the innate and the adaptive immune system. It was initially discovered as a key molecule in the regulation and prevention of autologous complement deposition on the cell surface^1^. Later, CD46 was found to be a potent co-stimulatory molecule inducing proliferation in T cells upon CD3/CD46 co-ligation^2^.

The CD46 molecule contains four short consensus repeats (SCRs), a serine, threonine, and proline rich region (STP region) followed by a transmembrane domain and a short cytoplasmic tail^3^. Alternative splicing of exons 7, 8, and 9 encoding the STP domains (denoted A, B, and C, respectively) and of exons 13 and 14 encoding the cytoplasmic tails 1 and 2 (CYT-1 and CYT-2) generates several CD46 isoforms, of which four are commonly expressed and designated BC1, BC2, C1, and C2, describing the included STP domain(s) and the cytoplasmic tail^4^.

Although these isoforms are usually co-expressed, tissue specific predominance of a specific isoform has been reported. In brain tissue C2 is the main isoform being expressed, whereas in the kidney and salivary glands BC2 is the most frequent isoform^5^. One study has examined the phenotypic expression pattern of CD46 isoforms in peripheral blood mononuclear leukocytes (PBMCs) in a larger population and found that 65% of the population preferentially expressed the BC isoforms, 29% had an equal distribution of the BC and the C isoforms and only 6% expressed mainly the C isoforms^6^. It was suggested that the phenotypic expression pattern is autosomal co-dominantly inherited. Peripheral blood leukocytes (PBLs) express both the CYT-1 and CYT-2 containing isoforms^7^, which have intracytoplasmic residues that are phosphorylated upon CD46 crosslinking^8^. This indicates that both cytoplasmic tails have the capacity for signal transduction.

The CD3/CD46 co-stimulation induces differentiation into a phenotype with regulatory capacity^9^. Although IL-2 does not modulate the overall expression of CD46^10^, it may control a change in cytokine profile, since CD3/CD46 co-stimulation in the presence of low amounts of IL-2 induces the secretion of IFNγ, whereas CD3/CD46 co-stimulation with increasing concentrations of IL-2 induces a switch to produce IL-10 through an intermediate step where the cells produce both IFNγ and IL-10. It was observed that only Jurkat cells overexpressing BC1, but not BC2, were capable of secreting IL-10 upon CD46 co-stimulation raising the possibility that CYT-1 might be involved^11^.

The binding of C3b to CD46 may enhance the uptake of nutrients and lead to an increased glycolysis and oxidative phosphorylation, which is important for the differentiation into Th1 cells^12^. Besides inducing a Th1-like response, CD46 also appears to be responsible for the intrinsic regulation of the contraction of this response. A coordinated induction and contraction of the Th1 response is dependent on a synchronized processing of the different cytoplasmic tails by presenilin/γ-secretase (P/γS). Initially, P/γS cleaves the CYT-1 tail in order to activate the T cells and induce a cytokine response, and subsequently P/γS cleaves the CYT-2 tail, which leads to a contraction of the proliferation and cytokine production^10^. Thus, the different cytoplasmic tails of CD46 may have distinct functions in induction and contraction of an effective immune response induced by CD46 co-stimulation.

The CD46-induced IL-10 production from CD4^+^ T cells is reduced in patients with multiple sclerosis (MS)^13^,^14^. This has also been observed in an animal model of MS, experimental autoimmune encephalitis (EAE) in cynomolgus monkeys. CD4^+^ T cells from these EAE monkeys are impaired in production of IL-10, but not IFNγ upon CD46 co-stimulation^15^. Dysfunctional CD46 signalling might also be implicated in the pathogenesis of rheumatoid arthritis (RA), where CD4^+^ T cells have a defect in the shutdown of IFNγ production following CD46 co-stimulation. Consequently, the switch to IFNγ^−^IL-10^+^ cells was impaired, which reduced the contraction of the Th1 response, resulting in an increased production of IFNγ^11^. A dysfunctional CD46 signalling in MS and RA could in part be caused by a defect in the regulation of CD46 isoform expression during the immune response, and notably such epigenetic changes are not caught in a genetic screen of these diseases.

Recent data demonstrate that the 5′ splice sites of exons 7 and 8, which determine the BC or C isoforms, are defined by base paring to U1 small nuclear RNA, whereas regulation of exon 13 inclusion, which gives rise to CYT-1 isoforms, is under control of multiple exonic and intronic splicing enhancers and silencers. Of these, SRSF1 and PTBP1 act as repressors of exon 13 inclusion, which would favour CYT-2 isoforms^16^.

It is unknown how these isoforms are expressed on immune cells. Previously, studies on 18 healthy individuals found that CYT-2 isoforms were more prevalent than CYT-1, but it was not examined whether this was due to BC2 or C2^13^. Other studies have suggested a dominance of CYT-2, but this have been done using antibodies to the cytoplasmic tails or non-quantitative PCR, which do not allow comparisons between the relative frequencies of the CYT tails, nor does it investigate the isoforms being expressed^10^,^12^.

Considering that splicing into specific CD46 isoforms are critical for regulating the immune response, we performed a comparative analysis of the expression of the individual CD46 isoforms on subsets of immune cells. By conducting longitudinal analysis of activated CD4^+^ T cells, we examined the previous hypothesis that the expression of CD46 isoforms is inherited.

Our data suggest that the splicing of CD46 is non-random and that exclusion of exons 7–8 and exon 13 is associated. Control of CD46 isoforms by epigenetic factors may allow temporarily and rapid regulatory changes during an immune response and may be an underestimated mechanism of local immune regulation.

## Results

### Development of a real-time PCR assay to detect the expression of individual CD46 isoforms

**Figure 1:**
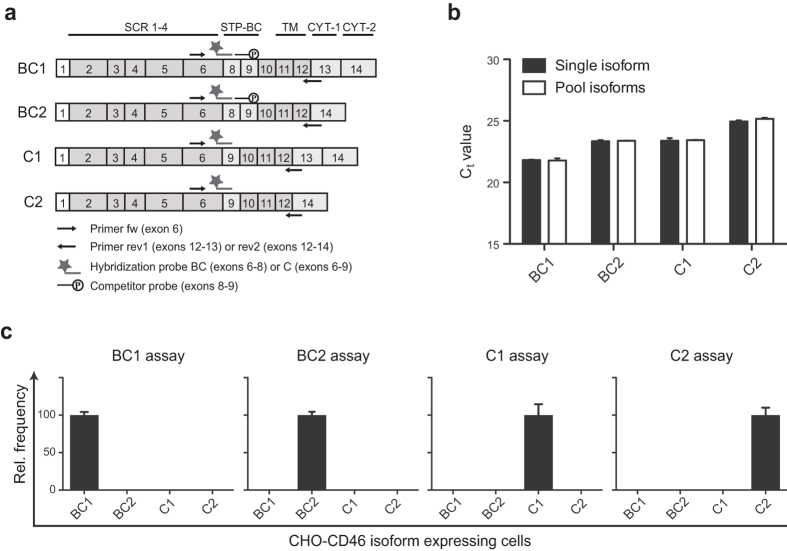
Design and test of a CD46-isoform real-time PCR assay. (**a)** Schematic representation of the mRNA for the four CD46 isoforms BC1, BC2, C1, and C2. The localization of primers and probes in the CD46-isoform real-time PCR assay is indicated on the figure. The fw primer is located in exon 6 and amplifies all isoforms, whereas the rev primers are located in either the exons 12-13 junction amplifying CYT-1-expressing isoforms or in the exons 12–14 junction amplifying the CYT-2-expressing isoforms. The hybridization probe detecting the BC isoforms is located in the exons 6–8 junction and the probe detecting the C isoforms is located in the exons 6–9 junction. (**b**) Efficiency of the real-time PCR assay. Equivalent amounts of cDNA from CHO cell lines each stably expressing the ORF from each of the four CD46 isoforms (BC1, BC2, C1 and C2) was mixed with cDNA from CHO wt cells (“Single isoform”) or cDNA from CHO cells expressing each of the other isoforms (“Pool isoforms”) and analysed by the real-time PCR assay to test the efficiency of the assay for each of the single isoform upon presence of all the other isoforms. The y-axis indicates the Ct value for the isoforms. Error bars indicate +SEM of two technical replicates and the data is representative of two independent experiments. (**c**) Specificity of the CD46-isoform real-time PCR assay. The four CHO cell lines each stably expressing the ORF from each of the four CD46 isoforms (BC1, BC2, C1, and C2, as indicated on the x-axes) were analysed by the real-time PCR assay to test for the specificity of the assay. The y-axes indicate the relative frequency of the four isoforms. Error bars indicate +SEM of three technical replicates.

Until now it has not been possible to quantify the individual expression of each of the four common CD46 isoforms in a given sample due to the high similarity between the isoforms and the lack of a unique genomic sequence that distinguishes these isoforms from each other. To quantitatively analyse and compare the expression of the four isoforms BC1, BC2, C1, and C2, we developed a real-time PCR assay that allowed us to distinguish between all four isoforms. A schematic diagram illustrating the design of the assay is shown in Fig. 1a. The assay includes a forward (fw) primer located in exon 6 that amplifies all four isoforms, reverse primers (rev) located in either the exons 12–13 junction or the exons 12–14 junction to distinguish between mRNA for CYT-1 (rev1) and CYT-2 (rev2) respectively. In addition, fluorescently labelled hybridization probes located in either the exons 6–8 junction to detect mRNA for BC isoforms (probe BC) or in the exons 6–9 junction to detect mRNA for the C isoforms (probe C) were used. Primers and probes are listed in table 1. Combining the fw primer with the different rev primers and the different hybridization probes allows analysis of mRNA for each of the common CD46 isoforms separately. For instance, to detect the relative mRNA expression of the BC1 isoform, the fw primer was mixed with rev1 and probe BC.

Since the exons 6–9 junction has a high similarity to the exons 8–9 junction, a competitor probe with a 3′phosphate was included in the assay for detecting C1 and C2 to avoid an unspecific signal from probe C binding to the exons 8–9 junction. A 20-fold excess of the competitor probe relative to the fluorescently labelled probe was proven to prevent unspecific signals without affecting the PCR reaction of the correct isoform or the reference gene (data not shown). The assay was performed using multiplex PCR with the reference gene peptidyl-prolyl cis-trans isomerase B (PPIB). The efficiency of the assay for the different isoforms as well as PPIB was analysed using a 10-fold serial dilution of plasmids containing each of the different isoforms mixed with purified PCR products for PPIB. The efficiency of amplification in each of the isoform assays was between 99.2–101.7% within a range from 18 to 35 C_t_ values and the efficiency of PPIB amplification in each of the assays was between 97.7 and 103.2% in the same C_t_-value range (data not shown).

Since all human cells express a mixture of the isoforms, we generated CHO cells ectopically and stably expressing each of the individual isoforms. To further validate that the efficiency of the assay for each of the different isoforms was not affected by the presence of all four isoforms in the samples, equivalent amount of cDNA from CHO cells expressing each of the distinct isoforms was mixed with either cDNA from CHO wt cells or cDNA from CHO cells expressing the other isoforms and analysed by the real-time PCR assay (Fig. 1b). This demonstrated that the Ct value for each of the distinct isoforms was not changed upon the presence of the other isoforms in the sample.

Moreover, PCR reactions on CHO cell lines stably expressing with BC1, BC2, C1, or C2 demonstrated that the real-time PCR assay was able to identify the correct CD46 isoform and was specific, since an isoform was not amplified with primers for the other isoforms (Fig. 1c).

### CD46 isoform expression in CD4^+^ T cells from healthy individuals

**Figure 2:**
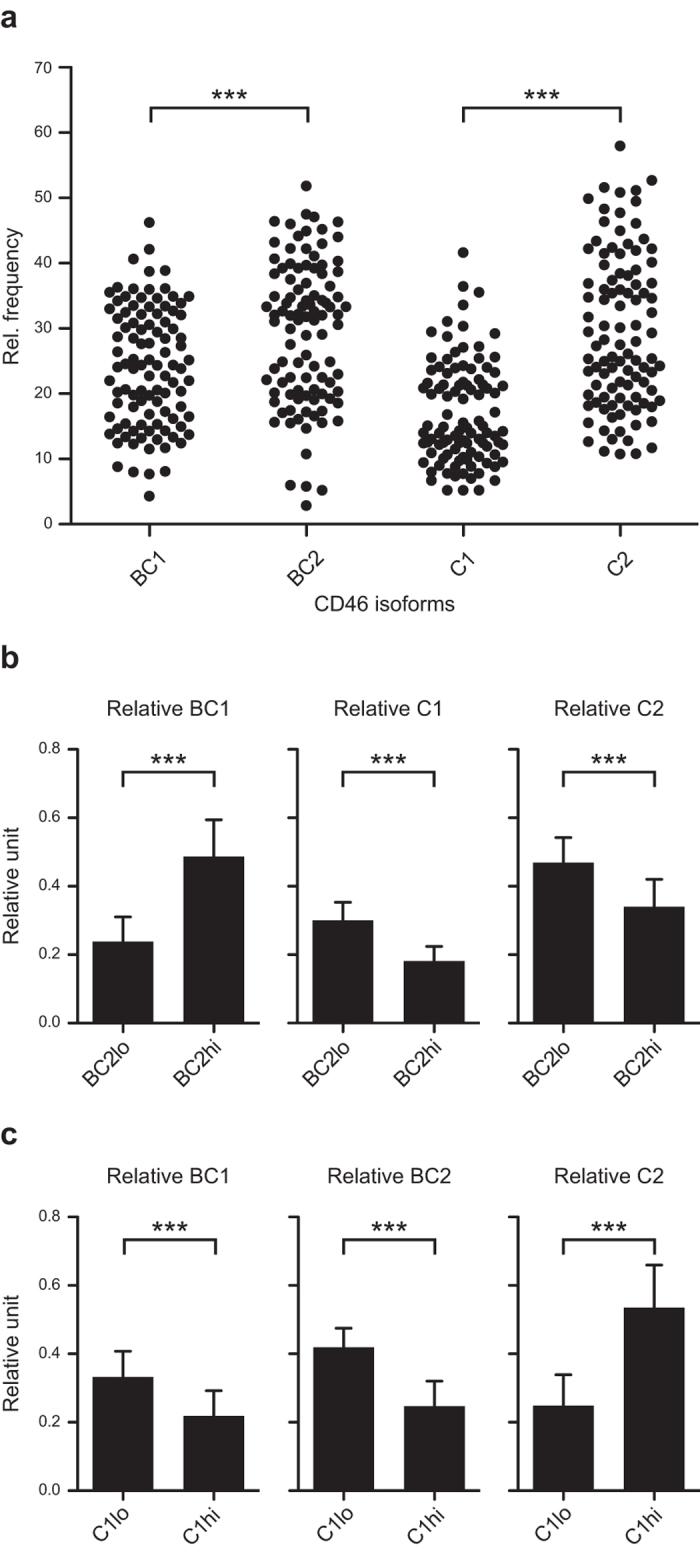
Distribution of different CD46 isoforms in the human population. (**a)** CD4^+^ T cells were isolated from 100 consecutive blood donors (median age 42 years, range 20–64 years, equal gender distribution). The relative mRNA expression of the different CD46 isoforms was measured by real-time PCR and is indicated as a relative frequency. Each dot represents the mean of 3 technical replicates for a given individual for a given isoform. ***indicates P < 0.0001 (Wilcoxon matched pairs signed rank test). (**b**) Analyses of isoform co-expression in BC2 low (BC2lo) and BC2high (BC2hi) expressing CD4^+^ T cells separated by the relative frequency of 28% according to the data in (**a**). Data are shown as relative units defined by the relative expression of the given isoform divided by the summarized relative expression of BC1, C1 and C2. (**c)** Analyses of isoform co-expression in C1 low (C1lo) and C1high (C1hi) expressing CD4^+^ T cells separated by the relative frequency of 18% according to data in (**a**). Data are shown as relative units defined by the relative expression of a given isoform divided by the summarized relative expression of BC1, BC2 and C2. For (**b,c)** data are represented as mean + SD and ***indicates P < 0.0001 (Mann Whitney test).

Previous analyses of CD46 isoform expression in a larger population have only been focusing on BC versus C isoforms, because of the lack of a quantitative assay to detect and compare all four isoforms of CD46. To examine the relative expression of CD46 isoform mRNA in healthy individuals, we isolated CD4^+^ T cells from 100 consecutive blood donors (age 20–64, median age 42, with an equal gender distribution). The relative mRNA expression from each of the isoforms was characterized using the assay described in Fig. 1, and the relative frequency of a specific isoform was defined as its expression compared with all four isoforms. This demonstrated that the CD46 isoforms are widely but dichotomously distributed in the population (Fig. 2). The most predominant isoforms are BC2 and C2, both containing the CYT-2 intracytoplasmic tail. This suggests that CD4^+^ T cells express both CYT-1 and CYT-2 isoforms, with a dominance of the CYT-2 isoforms (Fig. 2a).

To examine whether the isoforms were randomly expressed, the BC2 distribution was divided into BC2 low (BC2lo) and BC2 high expression (BC2hi) separated by a relative frequency of 28% (Fig. 2a). When the co-expressed isoforms were analysed, the presence of C1 and C2 reversely correlated with the expression of BC2, whereas the expression of BC1 directly correlated with BC2 (Fig. 2b). Likewise, the distribution of C1 was separated by a relative frequency of 18% into C1lo and C1hi. A similar analysis, as performed for BC2, indicated that C1 reversely correlated with BC1 and BC2, but directly correlated with C2 (Fig. 2c). In addition, analyses of a possible linkage between the two splicing events were examined. The STP splicing favoured BC over C (p = 0.03, Wilcoxon matched pairs signed rank test). Despite this, C isoforms splice significantly more frequent to CYT2 than BC isoforms (p < 0.0001, Mann Whitney test), indicating that the conditions that favour exclusion of exons 7–8 (that is C over BC), also favours exclusion of exon 13 (that is, CYT-2 over CYT-1). This indicates that splicing leading to exclusion of exon 8 is associated with exclusion of exon 13.

### Phenotypic expression patterns on subpopulations of PBMCs

**Figure 3:**
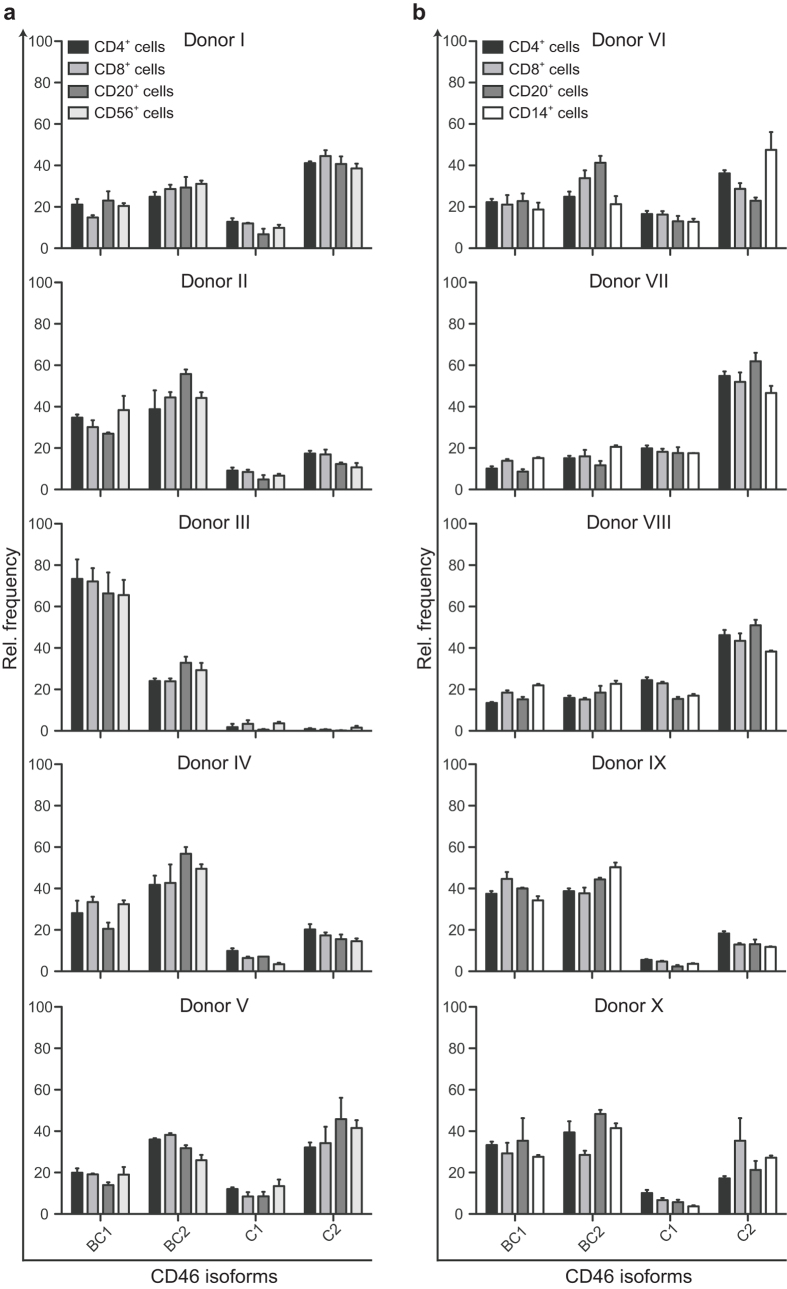
Phenotypic expression profiles of CD46 isoforms on main subpopulations of PBMC. PBMC from 10 different donors were sorted in subpopulations based on expression of the following surface markers: CD4^+^, CD8^+^, CD20^+^, and CD56^+^ (**a**) or CD14^+^ (**b**). From each of the populations mRNA was extracted and the relative expression of each of the different CD46 isoforms was analysed by real-time PCR. The expression of each of the different isoforms is shown as a relative frequency. The data are represented as mean + SEM of three technical replicates.

Previous studies on the expression of the individual CD46 isoforms in different tissues have been examining a heterogeneous PBMC population, although it comprises different cells with highly distinct functions in the immune system. PBMCs can be divided into monocytes and four main lymphocyte populations: CD4^+^ and CD8^+^ T cells, B cells, and NK cells. To address whether these different subsets express specific patterns of CD46 isoforms, PBMCs from 10 healthy donors were sorted into separate populations: CD4^+^, CD8^+^, CD20^+^, and CD56^+^ (Fig. 3a) or CD14^+^ (Fig. 3b) and analysed for the relative expression level of the different CD46 isoforms. For each individual the isolated subsets expressed the same pattern of isoforms. However, clear differences in expression patterns were observed among different individuals as represented in donors I, II, III, and VII. Donor I, V, VI, and X had a phenotypic pattern with BC2 and C2 being dominantly expressed, and with more or less equal frequencies of BC and C isoforms. Donor II, IV and IX had a phenotypic pattern with a dominant expression of BC1 and BC2. Donor III had a highly dominant expression of BC1 and almost no expression of C1 and C2, whereas donors VII and VIII had a C2-dominated expression. This demonstrated that despite the inter-individual differences in the pattern of expression, the intra-individual pattern was highly similar among the different subpopulations of PBMCs.

**Figure 4:**
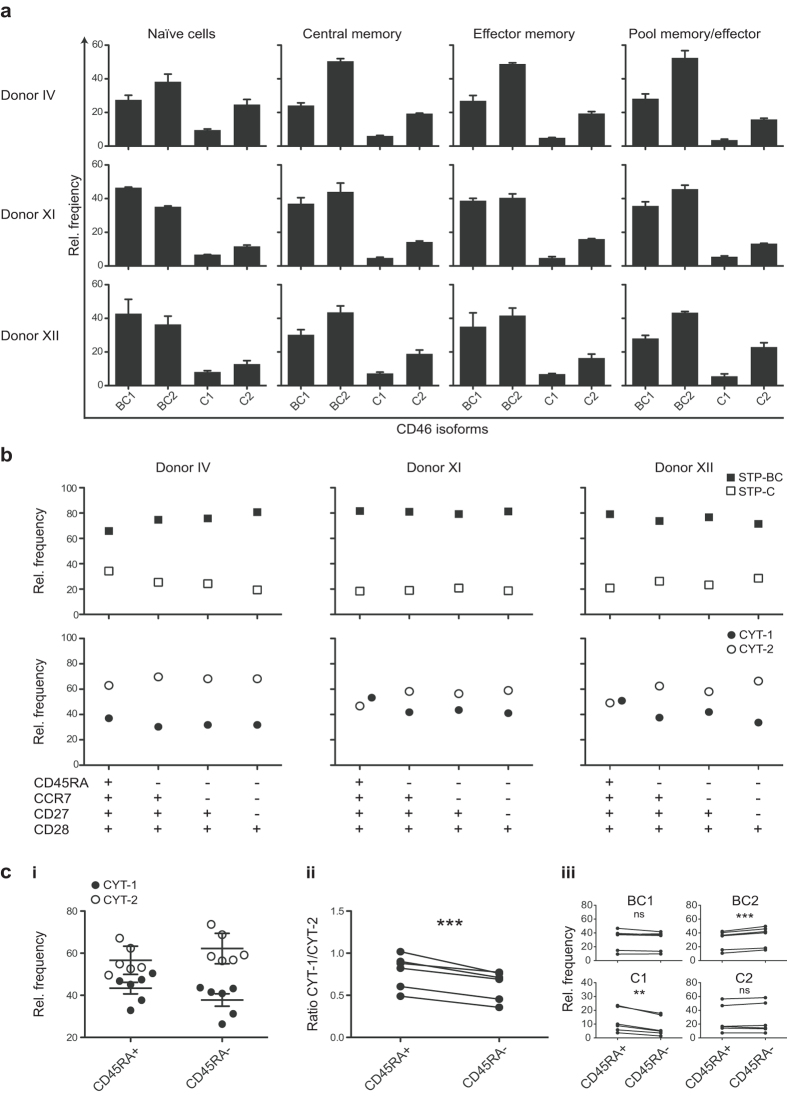
The frequency of CYT-2-containing isoforms in naïve and memory/effector CD4^+^ T cells. CD4^+^ T cells from three different donors were sorted into different subpopulations: Naïve (CD45RA^+^CCR7^+^CD27^+^CD28^+^), central memory (CD45RA^−^CCR7^+^CD27^+^CD28^+^), effector memory (CD45RA^−^CCR7^−^CD27^+^CD28^+^), and a pool of memory and effector (CD45RA^−^CCR7^−^CD27^−^CD28^+^) and subsequently assessed by real-time PCR for expression of the CD46 isoforms. The data are shown as a relative frequency. (**a)** mRNA expression of each of the separate CD46 isoforms in the different subpopulations indicated above from the three different donors. Data represent mean + SEM of three technical replicates. (**b)** Summarized relative frequency of BC and C containing isoforms (upper panel) or CYT-1 and CYT-2-containing isoforms (lower panel) in the different subpopulations shown from the same three donors as in (**a**). (**c)** CD4^+^ T cells from 6 different donors were sorted into either naïve (CD45RA^+^) or previously activated (CD45RA^−^) cells. mRNA was extracted and the relative mRNA expression of the different CD46 isoforms was assessed by real-time PCR. **ci)** Summarized relative frequency of the CYT-1 and CYT-2-containing isoforms in either CD45RA^+^ or CD45RA^−^ populations from the six donors. Mean values ± SD of CYT-1 and CYT-2 of the 6 donors are represented. (**cii)** Ratio of CYT-1/CYT-2 in all donors. (**ciii)** Change in relative frequency of each of the different isoforms in CD45RA^+^ compared with CD45RA^−^ populations. **indicates P < 0.01 and ***indicates P < 0.001 (paired t-test), ns = not statistically significant.

The subpopulations characterized in Fig. 3 comprise a mixture of both naïve and previously activated cells. We speculated that if previously activated cells gained more specialized functions, they might change their pattern of CD46 isoforms. To further address this, CD4^+^ T cells were sorted into different subpopulations of naïve, central memory, effector memory and pooled effector and memory cells based on differential expression of CD45RA, CCR7, CD27 and CD28. Based on these experiments, it appeared that the phenotypic expression pattern was similar in the different subpopulations (Fig. 4a).

Because previous data suggest that the different cytoplasmic tails of CD46 might have distinct functions in induction and contraction of an effective immune response, we decided to further examine the distribution of the frequencies of CYT-1 and CYT-2. From the summarized expression of either CYT-1 or CYT-2-containing isoforms, it appears that CYT-1 was expressed at a higher frequency in the naïve CD4^+^ T cells (defined by CD45RA^+^CCR7^+^CD27^+^CD28^+^) compared with the subpopulations of memory and effector cells (Fig. 4b). This difference was most pronounced for donor XI and XII, who had a frequency of about 50% for both CYT-1 and CYT-2 in the naïve CD4^+^ T cells, whereas the balance between CYT-1 and CYT-2 was shifted towards CYT-2 in the remaining subsets (approximately 40/60). In donor IV even the naïve CD4^+^ T cells had a frequency of about 60% of CYT-2-containing isoforms, nonetheless this increased slightly to about 70% in all the subpopulations that had markers of being previously activated. Hence, this donor showed the same trend as was seen for donor XI and XII (Fig. 4b).

It thus appears that CD4^+^ T cells expressing markers of being previously activated during an immune response have an increased expression of CYT-2-containing isoforms. To address this hypothesis, we sorted CD4^+^CD45RA^+^ and CD4^+^CD45RA^−^ cells from 6 different donors and analysed their expression of the CD46 isoforms by real-time PCR. A slight increase in CYT-2-containing isoforms in the CD45RA^−^ population compared with CD45RA^+^ population was observed for all donors (Fig. 4c,i). A statistically significant decrease of the CYT-1/CYT-2 ratio in the CD45RA^−^ population was consistent with the relative increase in the frequency of CYT-2 (Fig. 4c,ii). The overall increased frequency of CYT-2-containing isoforms in previously activated cells resulted mainly from an increased frequency of BC2 and a decreased frequency of C1, whereas the frequencies of BC1 and C2 were unchanged (Fig. 4c,iii). Importantly, the relative increase in CYT-2-containing isoforms demonstrates that the balance between the isoforms changed upon activation.

### Modulation of CYT-1 and CYT-2 expression upon activation of CD4^+^ T cells

**Figure 5:**
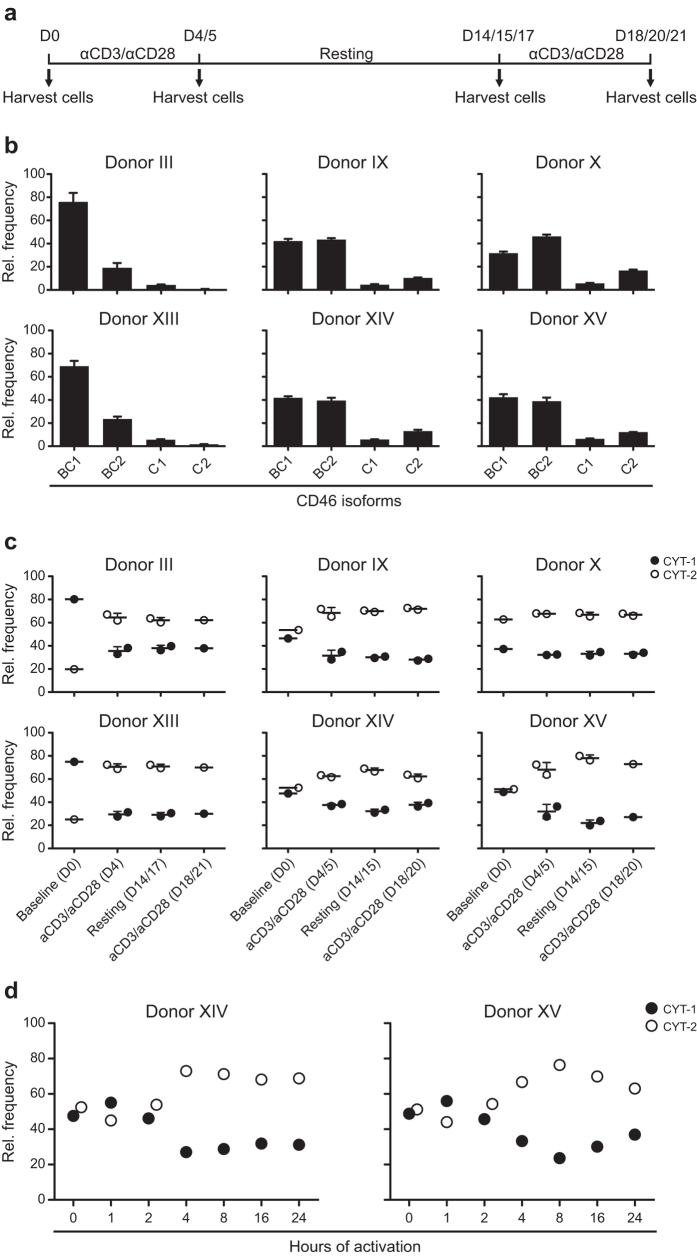
Expression of CD46 isoforms in *in vitro*-activated CD4^+^ T cells. CD4^+^ T cells were isolated from six different donors and activated with 2 μg/ml plate-bound anti-CD3/anti-CD28 for several rounds. (**a)** Culture scheme of the experiment for panel b and c. Arrows indicate time-points where an aliquot of the cells was harvested. (**b)** mRNA was extracted from the isolated CD4^+^ T cells from each donor immediate after the isolation, and the relative expression level of the different isoforms was determined by real-time PCR. The data are represented as a relative frequency of a given sample and provide a baseline for the stimulation experiment. Error bars indicate + SEM of three technical triplicates. (**c)** Summarized relative frequency of CYT-1 and CYT-2-containing isoforms in the different donors at baseline and at the following time-points after activation and subsequent resting of the cells. The mean ± SEM of two separate wells for each donor at each given time-point is illustrated. (**d)** Summarized relative frequency of CYT-1 and CYT-2-containing isoforms in two different donors at baseline and upon early activation of the cells during a period of 24 hours.

To directly examine the impact of activation on CYT-1 and CYT-2-expressing isoforms, several rounds of *in vitro* activation of isolated CD4^+^ T cells was performed with anti-CD3/anti-CD28 in the presence of 20 U/ml rIL-2. Bulk CD4^+^ T cells from six different donors representing different ratios of CYT-1/CYT-2 were isolated and activated according to the scheme in Fig. 5a. At different time-points during the culture period, an aliquot of the cells was harvested and analysed for the mRNA expression of the different CD46 isoforms. Initially, the baseline expression of CD46 isoforms for each of the donors was examined (Fig. 5b) demonstrating that they all had a distinct pattern. Donor III and XIII were highly dominant for BC1 and had almost no expression of C1 or C2, donor IX, XIV, and XV had an almost equal expression of CYT-1 and CYT-2 and was highly BC dominant, and donor X was dominant for CYT-2-containing isoforms. Upon anti-CD3/anti-CD28 activation for 4 or 5 days (Fig. 5c), the relative expression of CYT-2 increased to about 70% of the total expression in all six donors, despite their difference in frequency of CYT-2 at the baseline level. This switch in splicing of CYT-tails could be detected as early as 4 hours after activation (Fig. 5d) and was stable during the resting phase of the culture period, although the cells did not receive additional anti-CD3/anti-CD28 stimulation, and it was not further modulated upon the second re-activation of the cells (Fig. 5c).

**Figure 6:**
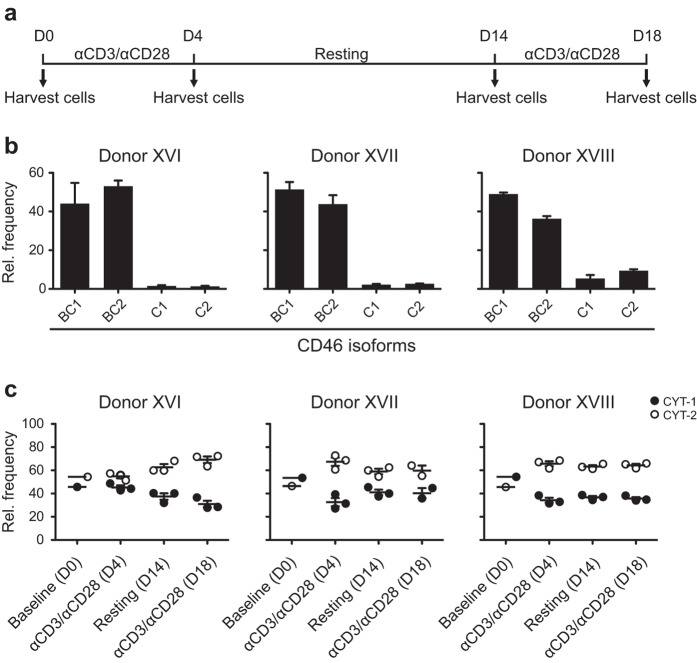
Expression of CD46 isoforms in *in vitro*-activated naive CD4^+^ T cells negatively isolated from CBMCs. Naïve CD4^+^ T cells from three different samples of CBMCs were activated with 2 μg/ml plate-bound anti-CD3/anti-CD28 for several rounds. (**a)** Culture scheme of the experiment. Arrows indicate time-points where an aliquot of the cells was harvested. (**b)** mRNA was extracted from the isolated naïve CD4^+^ T cells from each donor immediate after the isolation, and the relative expression level of the different isoforms was determined by real-time PCR. The data are represented as a relative frequency of a given sample and provide a baseline for the stimulation experiment. Error bars indicate + SEM of three technical triplicates. (**c)** Summarized relative frequency of CYT-1 and CYT-2-containing isoforms in the different donors at baseline and at the following time-points after activation and subsequent resting of the cells. The mean ± SEM of three separate wells for each donor at each given time-point is illustrated.

To further address the behaviour of naïve CD4^+^ T cells, these cells were isolated from cord blood mononuclear cells (CBMCs). The baseline level of CYT-1 and CYT-2 as well as the frequency following activation with anti-CD3/anti-CD28 was performed according to the schedule shown in Fig. 6a. Similar to the data shown in Fig. 5, isolated naïve CD4^+^ T cells from each donor expressed an increase in CYT-2 upon activation (Fig. 6b,c).

## Discussion

It has previously been suggested that the phenotypic expression pattern of CD46 isoforms is genetically determined^6^,^17^. The four common isoforms of CD46 have, however, very high similarity, for which reason the expression of each of them separately is very difficult to analyse. Based on the protein molecular mass it is possible to distinguish between the BC and C isoforms by Western blotting. The BC isoforms have a mass of about 65 kDa whereas the C isoforms have a mass of about 55 kDa^18^, but it is not possible to distinguish between CYT-1 and CYT-2-expressing isoforms solely based on mass in a Western blot. Discrimination between CYT-1 and CYT-2 has been achieved previously by PCR amplification assay^13^ that did not, however, discriminate between BC and C isoforms. Another widely used approach to analyse the expression level of each of the isoforms separately has been amplifying all isoforms by PCR and subsequently separating the PCR products on an agarose gel^4^. By this method it is possible to separate the expression of all four isoforms at the same time, but it does not provide a very accurate measurement of the difference in expression levels between the isoforms. To be able to quantify the differences in expression level, we therefore developed a real-time PCR assay that discriminated between mRNA of the separate isoforms.

In addition to alternative splicing, the CD46 transcripts might be regulated by transcription speed and non-sense-mediated mRNA decay^16^. For our studies, we assume that the mRNA level of each of the isoforms roughly correlates with the expression at the protein level. This is based on observations by Post *et al.* who used band intensities on an agarose gel to demonstrate that mRNA levels of the different CD46 isoforms correlated with the protein phenotype^4^. Furthermore, the absence of recycling of CD46 between the surface and intracellular storage compartments in PBMCs and several lymphoid cells lines^19^ support this assumption, at least for lymphocytic cells.

The quantitative measurement of the expression of BC1, BC2, C1, and C2 allowed comparisons between the individual isoforms. This indicated that the distribution of a given isoform did not follow a Gaussian distribution, but rather separated in two populations, which we arbitrarily defined as a high and low expression. Noticeably, these levels refer to the relative frequency of the individual isoform and not the absolute level of the isoform. The relative frequency of an isoform may be more significant than the absolute level considering the assumption that different isoforms exerts opposing effects within the cell. Thus, an imbalance in CD46 isoforms might be a potential co-factor contributing to disturbed immune regulation and disease development.

As is the case for most cells, CD4^+^ T cells also express all CD46 isoforms, although the relative distribution between the major four isoforms, BC1, BC2, C1, and C2, have not been examined in a larger population. Our analyses demonstrated a predominance of CYT-2 containing isoforms on cells with slightly but significantly more C2 than BC2, whereas CYT-1-containing isoforms were less frequent, but with slightly more BC1 than C1. Importantly, we found that the isoforms were not randomly paired. Splicing at STP favoured inclusion of exon 8 resulting in significantly more BC than C isoforms. On the other hand, when exon 8 was excluded to yield C isoforms they were significantly more often associated with exclusion of exon 13 and splicing to exon 14 (CYT-2) than was the case for BC forms. We demonstrated that high frequency of the BC2 isoform was inversely correlated with the presence of C isoforms. BC isoforms have been found to have higher affinity for C4b, but not C3b, than C forms, suggesting that it may matter for the immune protection whether an individual expresses BC rather than C^20^. Analyses of the splicing of exons 7–9 suggest that these exons have different inclusion efficiencies with exon 9 (C) as most efficient followed by exon 8 (B) and exon 7 (A)^16^. Although other cis-acting elements are involved in determining the exclusion of exon 8 and exon 7, our data suggest that inclusion of exon 8 is more frequent than exclusion of this exon.

Our observation that C2 and BC2 were the dominating isoforms within the population, demonstrate that on average the CYT-2-containing isoforms are the most prevalent on CD4^+^ T cells. This is in agreement with Astier *et al.* who previously described dominant CYT-2 expression in healthy individuals and MS patients^13^. Moreover, it is in agreement with the hypothesis of Astier and colleagues, who suggest that processing of CYT-1 is required to activate the T cell, whereas CYT-2 functions as a negative regulator to turn off the response^10^. Kolev *et al.* have furthermore reported an initial relative increase in CYT-1-containing isoforms upon activation of T cells^12^. Activation of T cells through co-stimulation of CD3-CD28 or CD3-CD46 produces intracellular C3b^21^,^22^, which by an autocrine mechanism may bind to CD46 and induce IL-10^22^. The autocrine function of C3b is thought to occur rapidly, and the expected later increase in CYT-2 may then function to curtail the response.

If the major function of CYT-2-containing isoforms is to convey a negative signalling, it may suggest that CYT-2 is important for preventing unwanted immune responses and might potentially explain the importance of a dominant expression of CYT-2 on immune cells. We confirm the previous observation of an activation-induced increase in CYT-2 expression, and demonstrate that within the population C2 is slightly but significantly more frequent than BC2. Moreover, we demonstrate that the upregulation of CYT-2 is maintained for a period of approximately 3 weeks, suggesting that the observed increase in CYT-2 is sustained during an immune response. This relative increase in CYT-2 was also observed when activating naïve CD4^+^ cord blood T cells *in vitro* with anti-CD3/anti-CD28 antibodies. Similar to the data on total CD4^+^ T cells, the phenotypes on the cord blood T cells were dominated by BC1 and BC2, which is a common phenotypic pattern. Noticeably, we did not find a successively increase in CYT-2 with age, which suggest that the activation-induced increase in CYT-2 is reversible to an epigenetically controlled steady-state level. Our demonstration of a similar CD46 isoform expression pattern within different immune subsets further supports this notion.

CD46 is a receptor for a number of microorganisms^23^, and a dominant expression of the presumed suppressive CYT-2-containing isoforms might have implications for the binding of microorganisms to their receptor. If all isoforms have same affinity for the pathogens that use CD46 as a receptor, the relative increased frequency of CYT-2 may cause pathogens to induce a negative signal into the host cell upon binding. If CD46-binding pathogens had a preference for some of the isoforms, a change in their expression may influence susceptibility to infections with the pathogen. However, whether or not all isoforms are functioning equally well as receptors for CD46-binding pathogens remains to be determined.

A few studies so far have addressed the question about expression of the different isoforms on different subsets of cells in the human body^5^,^24^. Our finding that the relative expression of the four most prevalent isoforms are similar on different subsets of the immune cells suggests that a genetic/epigenetic control maintain a certain pattern of expression within an individual, despite the fact that the expression is subject to modulation upon external stimuli. This indicates that the expression of CD46 isoforms is regulated in a complex manner, which may include genetically modified levels of transcription and splicing factors that determine the overall expression pattern. Given the emerging knowledge of the different functions of CD46 isoforms, it may be interesting to thoroughly correlate CD46 isoform patterns with diseases, particularly those of autoimmune nature. Previous studies have found an altered relative upregulation of CYT-2 in patients with multiple sclerosis^13^. However, it is not clear whether this is an epiphenomenon arising as a consequence of other aberrations in the immune regulation or whether it more profoundly characterizes a primary defect in patients with MS.

In summary, we have developed an assay that allows a comparison of the relative expression of the different CD46 isoform mRNAs and used the assay to deepen our understanding of the significance of CD46 splicing. Our data suggest that splicing at STP favours BC, but that C isoforms are significantly linked to CYT-2 splicing. Overall, the most frequent isoforms are CYT-2 containing. We demonstrate that the pattern of isoform expression has high interpersonal variance but low intrapersonal variance, since subsets of immune cells display the same expression profile. Noticeably, this profile can be modulated upon activation, with an altered CYT-1/CYT-2 balance between CD45RA^+^ and CD45RA^−^ cells. Since all the isoforms are simultaneously expressed on immune cells, fine-tuning in their relative abundance within an individual may be of importance in controlling the immune response. Although it is assumed that the different isoforms exert separate roles during the immune response, CD46 must be working in close collaboration with other regulatory proteins, since very different patterns of CD46 isoform expression can be identified among healthy individuals. The quantitative comparison between the isoforms allows future investigations into questions such as whether or not individuals with rare isoform patterns are associated with immune-mediated diseases or whether or not individuals with skewed isoform expression patterns have different susceptibility to infections with pathogens using CD46 as a receptor.

## Materials and Methods

### Ethical approval

Consecutive blood samples from 100 Caucasian donors were collected anonymously at the Blood Bank of the Department of Clinical Immunology, Aarhus University Hospital, with registration of only gender and age, according to the guidelines from the Danish Society for Clinical Immunology and the Ethical Committee on the use of donor samples for research purposes. Anonymous cord blood samples were obtained from the Department of Obstetrics and Gynecology, Aarhus University Hospital. Methods were carried out in accordance with the approved guidelines. Informed consent was obtained from all individuals.

### Cloning of the CD46 isoforms BC1, BC2, C1, and C2

The open reading frame (ORF) of each of the CD46 isoforms, BC1, BC2, C1, and C2 was amplified by PCR from the HCT-116 cell line or PBMCs using DreamTaq DNA polymerase (Thermo Scientific, USA) and 500 nM of the following primers (TAG Copenhagen, Denmark); fw: 5′-ATGGAGCCTCCCGGCCG, rev-13: 5′-GTCAGAGAGAAGTAAATTTTACTTCTCTGTGGGTC (for amplifying BC1 and C1), and rev-14: 5′-GTCAGCCTCTCTGCTCTGCTGG (for amplifying the BC2 and C2). The PCR products were purified from a 1% agarose gel (1g w/v agarose (Invitrogen, USA) dissolved in TAE (Tris-NaOH + 2% acetic acid + 1 mM EDTA) (Substrate department, AU, Denmark)). The 3′deoxyadenosine overhang generated by the Taq polymerase was used for ligation of the PCR products into the pcDNA3.1/V5-His-TOPO vector (Invitrogen, USA). The plasmids were cloned in TOP10 *E. coli* (Invitrogen, USA) according to the manufacturer′s instructions. The orientation and correct sequence of the ORFs were verified by Sanger sequencing (GATC, Germany) using the following primers (TAG Copenhagen, Denmark) covering the insert: T7 fw 5′-TAATACGACTCACTATAGGG, and BGH rev: 5′-TAGAAGGCACAGTCGAGG. BC1-, BC2-, C1-, and C2-ORFs were transferred from the pcDNA3.1/V5-His-TOPO vector to the pcDNA5/FRT/TO vector using the restriction enzymes HindIII (5′) and XhoI (3′) (New England Biolabs, UK) and the Quick T4 DNA Ligase (New England Biolabs, UK). The plasmids were cloned in TOP10 *E. coli* and the sequence of the isoforms verified by Sanger sequencing as described above.

### Generation of CHO cells stably expressing CD46 isoforms

Flp-In CHO cell (hereafter referred to as CHO) (kindly provided by S.K Moestrup) was maintained in Ham’s F-10 Nutrient Mix (Gibco, USA) supplemented with 10% heat-inactivated FBS (Sigma-Aldrich, USA), 10 mM HEPES (Substrate department, Aarhus University, Denmark), 2 mM glutaMAX (Gibco, USA), 100 U/ml penicillin, and 100 μg/ml streptomycin (Substrate department). The cells were detached from the bottom of the culture flasks using 0.05% (v/v) trypsin (Gibco, USA) dissolved in PBS supplemented with 1 mM EDTA, and diluted 1:10 in fresh media 2–3 times a week. The cells were cultured at 37 °C, 5% CO_2_. For generation of CHO cells stably expressing the ORFs of the CD46 isoforms, the cells were co-transfected with the pcDNA5/FRT/TO/CD46-ORFs and the plasmid pOG44 (Invitrogen, USA) encoding the Flp recombinase mediating the integration of the ORFs into the FRT site in the CHO cells. Prior to transfection, the CHO cells were plated to a confluence of about 85%. For each transfection, 2 × 10^6^ cells were washed in PBS and pelleted by centrifugation at 100 ×*g*. The cells were resuspended in 100 μl SF 4D-Nucleofector Solution (Lonza, Switzerland) and mixed carefully with 3.6 μg pOG44 and 0.4 μg pcDNA5/FRT/TO/CD46-ORFs. The samples were transferred to Nucleovettes and electroporation was performed with program DT-133 on the 4D-Nucleofector (Lonza, Switzerland). At 48 hrs post transfection the cells were diluted to a density of about 25% in cell culture media supplemented with 500 μg/ml hygromycin B (Sigma-Aldrich, USA). The media was changed 2–3 times a week until clear clone formations were observed. The Flp-In system permits integration into the same genomic loci in all cells generating isogenic cell lines and the clones for each of the isoforms, respectively, were pooled together. The CHO_CD46-isoform_ cell lines were maintained in Ham’s F-10 supplemented as described above, including 500 μg/ml hygromycin B.

### Isolation of PBMCs and CBMCs

Human PBMCs from buffy coats of healthy donors (The Blood Bank, Aarhus University Hospital, Denmark) or CBMCs from cord blood (kindly provided by N. Uldbjerg, Department of Obstetrics and Gynecology, Aarhus University Hospital, Denmark) were isolated with density gradient centrifugation using Ficoll-Paque PLUS (GE Healthcare Bioscience AB, Sweden) according to manufacturer’s instructions. The isolated cells were cryopreserved at a concentration of 10^7 ^cells/ml in 90% heat-inactivated FBS and 10% DMSO (Sigma-Aldrich, USA) and stored at −80 °C until use.

### Purification of CD4^+^ T cells

CD4^+^ T cells were isolated from PBMCs by negative selection using EasySep Human CD4^+^ T Cell Isolation Kit (Stemcell Technologies, France) according to manufacturer’s instructions. The isolated cells were applied to a magnet for an additional step to increase the purity of the cells. Naïve CD4^+^ T cells were isolated from CBMCs by negative selection using the EasySep Human Naïve CD4^+^ T Cell Enrichment Kit (Stemcell Technologies, France) according to manufacturer’s instructions. The purity of the isolated naïve CD4^+^ T cells was analyzed by flow cytometry using anti-CD4 FITC (clone 13B8.2, Beckmann Coulter, USA) and anti-CD45RA-APC (clone H1100, BD Biosciences, USA) and was > 94% for all samples. The isolated cells were resuspended in RPMI (Gibco, USA) supplemented with 10% heat-inactivated FBS, 10 mM HEPES, 2 mM glutaMAX, and 2.5 nM sodium pyruvate, or directly lysed in RNA lysis buffer (Qiagen, Germany or Macherey-Nagel, Germany).

### Fluorescence activated cell sorting

Three different multicolour Ab panels were designed for sorting of the subpopulations of PBMCs; Panel A for main PBMC populations: CD3-APC (clone SK7, BD Biosciences, USA), CD4-PE (clone 13B8.2, Beckman-Coulter, USA), CD8-FITC (clone B9.11, Beckman-Coulter, USA), CD20-PE-Cy5 (clone 2H7, BD Biosciences, USA), CD56-PE-Cy7 (clone HCD56, BioLegend, USA), and Fixable Viability Stain V450 (BD Biosciences, USA). For sorting of monocytes a similar panel was used with the omission of CD3-APC, CD56-PE-Cy7, and Fixable Viability Stain V450 and inclusion of CD14-PE-Cy7 (clone 61D3, eBioscience, USA) and LIVE/DEAD Fixable Near-IR (ThermoFisher Scientific, USA); Panel B for subsets of CD4^+^ T cells: CD4-FITC (clone 13B8.2, Beckman-Coulter, USA), CD27-PerCP-Cy5.5 (clone M-T271, BD Pharmingen, USA), CD28-Pe-Cy7 (clone CD28.2, BD Pharmingen, USA), CD45RA-APC (clone H1100, BD Biosciences, USA), CCR7-PE-CF594 (clone 150503, BD Horizon, USA), and LIVE/DEAD Fixable Blue (ThermoFisher Scientific, USA); and Panel C for sorting naïve and previously activated cells: CD4-FITC, CD45RA-APC, and LIVE/DEAD Fixable Near-IR (ThermoFisher Scientific, USA). Prior to the staining of the cells with Panel A or C, the PBMCs were thawed and washed two times in PBS + 2% FBS and resuspended to a concentration of 20 × 10^6^ cells/ml in PBS + 2% FBS. Prior to the staining with Panel B, the PBMCs were thawed and washed two times in PBS without Ca^2+^ and Mg^2+^ supplemented with 2% FBS and 1 mM EDTA and CD4^+^ T cells were isolated using the EasySep CD4^+^ T cell Enrichment Kit (Stemcell Technologies, France) according to manufacturer’s instructions. The isolated CD4^+^ T cells were resuspended to a concentration of 20 × 10^6^ cells/ml in PBS + 2% FBS. The cells were incubated with Abs for 30 min at 4 °C and subsequently washed two times in PBS + 2% FCS. Between 50,000 to 200,000 cells of interests were sorted on a 4-laser BD FACSAria III (BD Biosciences, USA) and subsequently lysed in RNA lysis buffer RLT (Qiagen, Germany). The cells were kept on ice or at 4 °C during the entire process of staining and sorting.

### RNA extraction and cDNA synthesis

Total RNA was isolated on spin-columns using either the Nucleospin RNA (Macherey-Nagel, Germany) or the RNeasy Micro Kit (Qiagen, Germany) for the sorted cells, according to manufacturer’s instructions. The samples were treated on-column with DNase for 15 min and 12 μl of the isolated RNA was used for cDNA synthesis using the QuantiTect Revers Transcription Kit (Qiagen, Germany). The cDNA was diluted 1:5 in RNase-free water prior to real-time PCR.

### CD46-isoform real-time PCR

Primers and probes used for real-time PCR are listed in table 1. The real-time PCR analysis of mRNA for expression of the different CD46 isoforms was done using Brilliant Multiplex QPCR Mastermix (Agilent Technology, USA) with different combinations of 200 nM CD46 primers and 75 nM CD46 probes. To assess mRNA for the isoforms C1 and C2, a competitor probe (Comp) was included at a final concentration of 1500 nM. The real-time PCR was performed in multiplex with 200 nM PPIB primers and 100 nM PPIB probe. The real-time PCR was performed in a Stratagene 3005 Mx Pro (Agilent Technology, USA) using the following thermal cycles: Initial denaturation at 95 °C for 5 min followed by 40–50 cycles of 94 °C for 30 sec, 55 °C for 30 sec, and 72 °C for 30 sec.

The expression level of each of the isoforms relative to PPIB was calculated using the 2^−∆Ct^ method. The relative frequency of a specific isoform was calculated as the percentage of the summarized relative expression level of all isoforms using the formula:


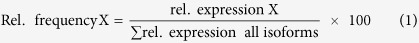


### T-cell activation

A 48-well cell culture plate was coated with 2 μg/ml anti-CD3 (clone OKT-3, eBioscience, USA) and 2 μg/ml anti-CD28 (clone CD28.2, eBioscience, USA). To each well 5 × 10^5^ isolated CD4^+^ T cells or 10^6^ isolated naïve CD4^+^ T cells were added together with 20 U/ml human rIL-2 (Roche Diagnostics, Germany) in a total volume of 500 μl RPMI supplemented with 10% heat-inactivated fetal FBS, 10 mM HEPES, 2 mM glutaMAX, and 2.5 nM sodium pyruvate. The cell culture media was refreshed with 20 U/ml rIL-2 every third day. Cells were transferred to a new uncoated 48-well plate for resting.

## Additional Information

**How to cite this article**: Hansen, A. S. *et al.* Non-random pairing of CD46 isoforms with skewing towards BC2 and C2 in activated and memory/effector T cells. *Sci. Rep.* **6**, 35406; doi: 10.1038/srep35406 (2016).

## Figures and Tables

**Table 1:** Oligonucleotides for real-time PCR.

Oligo^a^	Target	Sequence (5′–3′)	Modification	Length^b^	Tm^c^ (°C)
Fw	PPIB	TGTGGTGTTTGGCAAAGT		278	57.7
Rev		TGGAATGTGAGGGGAGTG			58.4
Fw	Exon 6, CD46	TGACAGTAACAGTACTTGGGA			57.2
Rev1	Exon 12/13, CD46	ATCAGTTAGGTATGTGCCTTTC		289/244^d^	57.0
Rev2	Exon 12/14, CD46	ACCATCTGCTTTCCCTTTC		286/241^d^	56.7
Probe	BC, CD46	CCAAAGTGTCTTAAAGTGTCGACTTCTTCCACTAC	5′-ROX^e^3′-BHQ2	NR^f^	66.5
Probe	C, CD46	AGTGTCTTAAAGGTCCTAGGCCTACTTACAAGC	5′-ROX^e^3′-BHQ2	NR	66.9
Probe	PPIB	AGAGCACCAAGACAGACAGCCG	5′-FAM3′-BHQ1	NR	66.4
Comp	Exon 8/9, CD46	GTGCCTCAGGTCCTAGGCCTACTTAC	3′-phosphate	NR	

^a^Oligo, oligonucleotides; indicated as Fw, forward primer; Rev, reverse primer; Probe, probe labeled with fluorescent dye and quencher; and Comp, complementary competitor oligonucleotide.

^b^Length, bases of amplified product.

^c^Tm predicted by the program CLC Main Workbench 6.

^d^Length of amplified BC/C isoforms using exon 6 forward primer.

^e^ROX analog CalFluor red 610.

^f^NR, not relevant.
